# Antecedents and Consequences of Streamer Trust in Livestreaming Commerce

**DOI:** 10.3390/bs13040308

**Published:** 2023-04-04

**Authors:** Bowen Tian, Jinye Chen, Jie Zhang, Wei Wang, Leibao Zhang

**Affiliations:** School of Business, Hangzhou City University, Hangzhou 310015, China

**Keywords:** livestreaming commerce, cognitive-affective-conative (C-A-C) framework, streamer trust, purchase intention, livestreaming value

## Abstract

Livestreaming commerce has become the mainstream of e-commerce in recent years. The key difference between livestreaming commerce and traditional e-commerce lies in the presence of the streamer. However, there are few studies that examine the significant role of streamer trust in the focal context. In our study, based on the cognitive-affective-conative (C-A-C) framework, we develop a research model to explore antecedents of streamer trust and its important role in influencing consumers’ purchasing behavior. Using the survey method, we find that (1) antecedents, including interactivity, informativeness, personal impulsiveness as well as the attitude toward livestreaming shopping are positively associated with streamer trust; (2) streamer trust is positively associated with consumers’ purchasing intention; (3) livestreaming value has significant moderating effects on interactivity and informativeness but not on personal impulsiveness and attitude toward livestreaming shopping. Both theoretical and practical implications are discussed.

## 1. Introduction

Livestreaming commerce has developed into a field that promises major investment potential. Compared with traditional e-commerce, livestreaming commerce, which shows products through real-time live video, has various advantages [1], such as timely interactions between consumers and streamers, straightforwardness, and vitality. The industry report of iMedia Research shows that the total scale of China’s livestreaming commerce industry is expected to reach RMB 2137.3 billion by 2025. Realizing the great potential of livestreaming commerce, researchers have been starting to pay attention to this field and dig into the great value of this new business model.

Several research topics could be identified from the current literature, such as pricing strategies [2], newly launched products, sales strategies under the contexts of long vs. short cooperation between retailer and MCN [3], manufacturers’ decisions regarding the open of livestreaming channel [4], optimal online channel structure for firms considering livestreaming shopping [5], impacts of streamers’ linguistic styles on sales performance [6], as well as the product brand extension [7]. From these streams of study, we typically realize that very interesting findings have been uncovered from prior innovative studies. However, to the best of our knowledge, little has been discussed regarding the significant role of trust in this new type of research context. 

Although we may recognize that trust has been widely studied in the past two decades in information systems research [8,9], electronic commerce [10,11] as well as marketing [12], it has to be realized that trust transfers its unique meanings varying along e-commerce context, and it is typically represented differently in different stages. Specifically, in the very early stage of e-commerce, which is called “platform-based e-commerce”, we may understand that website design signals the transfer of trust to potential consumers, such as the logo of a famous brand like Yahoo [13] or the perceived website quality [14]. Along with the development of e-commerce, new styles emerge when platform-based e-commerce is no longer the mainstream of e-commerce, but instead, “social-network-based commerce” is. In the new age of e-commerce, trust is typically represented by new styles, such as online review information [15] and sale volumes [16], where consumers’ purchasing behavior is greatly impacted by these types of information. Later, with the further development of e-commerce, the new style called “livestreaming e-commerce” came. In this focal stage of e-commerce, it is widely acknowledged that the focal information, such as the sales volume, online review volume, as well as the design of websites, do not work again. Because, in our daily life, we may realize that these types of information are not always shown in the livestreaming commerce context, but instead, a streamer’s show or live show guides potential consumers’ purchasing behavior. This leads us to propose the following questions: How does trust work in this focal context? What are the main antecedents, reviews, sales, or others? Does trust influence consumers’ final purchasing intention? 

As a matter of fact, in livestreaming commerce, a streamer is regarded as the opinion leader and is usually concerned by consumers [17]. A streamer plays a connection role between the product and the audience. The reputation of a streamer affects the audience’s perception of the product. The trust established between live audiences and the streamer is easily enhanced through the streamer’s good reputation [18]. Therefore, to fill this significant research gap in the current literature and bring along the above questions, this study, as one of the very initial attempts, tries to uncover the important role trust plays in livestreaming commerce through the identification of its antecedents and consequences. Drawing on the cognitive-affective-conative (C-A-C) framework and the related literature, several hypotheses are proposed and tested. This study contributes to the current knowledge in the following facets. First, to the best of our knowledge, this is a very initial study exploring the significant role of trust and revealing its antecedents and consequences in the context of livestreaming commerce, thus extending the present literature. Second, although previous studies have pointed out that trust should be impacted by several factors varying alone its research context, there is little discussed in the livestreaming context, and it is scantily understood regarding the different dimensional formation. This study from both the cognitive and affective dimensions fills this gap in a complementary way. Third, as with most current studies, moderation identification is always contributable to the underlying mechanism. This study explores the livestreaming value that moderates the effects of several antecedents on streamer trust building. 

The study is presented as follows. In Section 2, we review the related literature on livestreaming commerce and the C-A-C framework. Section 3 presents a conceptual model with hypotheses. Section 4 presents the research methodology, model testing, and analysis results for our proof-of-concept model. Finally, Section 5, Section 6 and Section 7 summarize our research contributions, limitations, and potential future research directions.

## 2. Literature Review

### 2.1. Livestreaming Commerce

As an increasingly important part of the livestreaming industry, livestreaming commerce is characterized by real-time interaction between the streamer, the audience, vivid product demonstration, and purchase behavior [1,19,20,21]. The interaction between the streamer and the audience plays a key role in the user experience because it promotes the flow of cognition and affection, making it possible to establish a solid and stable interpersonal relationship between the streamer and the audience [22,23]. Some previous studies have studied the characteristics of consumers and the impact of psychological factors in the shopping process from consumers’ perspective [24], and some studies have discussed the characteristics of streamers and the interaction between streamers and audiences from the perspective of streamers [20]. However, the current research has not yet thoroughly studied the antecedents and consequences of streamers. This provides a new perspective for the research of livestreaming commerce. 

Some research focuses on the impact of consumers’ personal characteristics on purchasing behavior. For example, Zhang et al. [25], upon investigating impulse buying behavior in livestreaming commerce, added personal impulse as a control variable and demonstrated that, in livestreaming commerce environments, impulsive buying is driven by affective state (i.e., emotional intensity) rather than cognitive state (i.e., perceived risk). Wu et al. [26] found that when consumers make purchases online, although personal impulse had a positive impact on their impulse purchases, it did not moderate the impact of perceptual arousal. In the study of Chen et al. [27], habits are found to reinforce the impact of product uncertainty on purchase intention precisely because the convenience of product search meets consumers’ preferences, and their interaction during livestreaming yields an overall happy experience. Eventually, a habitual dependence is formed, and this habit then regulates the relationship between product quality uncertainty and purchase intention.

External influences comprise the focus of most prior research. In the work of Wongkitrungrueng and Assarut [28], it was found that sellers can provide consumers with cognitive value by providing livestreaming content and services that consumers are interested in [21]. Consumers may, therefore, be grateful and exhibit reciprocal behavior with sellers because of the non-sales content provided by livestreaming for free. An empirical study by Kang et al. [29] found that interactivity has a curved relationship with audience participation behavior in livestreaming and that it, more precisely, increases the interaction between audiences and streamers will promote audiences’ willingness to purchase and actively send gifts. Chen et al. [27] also found that information is the core element that drives consumers’ product purchases; thus, if the product information is ambiguous, it will create a complex shopping process for consumers.

The concept of trust also comprises an important research direction, and studies have found that when consumer trust in streamers increases, sellers are capable of expanding their product portfolio [21]. Zhang et al. [30] found that trust (including trust in streamers and products) is key to audiences’ continued livestreaming; the study also found that social and technological drivers have a positive impact on consumer trust in livestreaming and that trust in streamers mediates between interactivity, IT delivery and trust in products.

Research on livestreaming commerce is still in its infancy, as most studies thus far have offered descriptions of livestreaming and consumer behavior (see Table 1). Based on the C-A-C framework, this paper examines consumers’ purchase intentions by studying their internal influences and external influences, both of which generate trust in streamers.

### 2.2. C-A-C Framework

Research in the field of psychology can be categorized into three segments: cognition, affection, and conation [33,34,35]. McDougall argues that the purest instinctive behavior can be adequately described through the lens of these three aspects of the mental process, each of which relates to the behavior of something, from the realization of feelings to the execution of the act. Cognition is the process of knowing and understanding; it encompasses the reception of information and its processing [36]. Affection refers to the emotional interpretation of information or things; in other words, it pertains to how people feel about the information they perceive [36]. Meanwhile, conation is the connection to behavior on a cognitive and affective basis; it can be described as a person’s behavioral intention or the subjective probability that they will use an information system [36,37]. The progression from cognition to affection to conation is connected in a universal way to the outside, the system, and the senses. An important marker of cognition is its representation, and although the senses that provide this characteristic are mostly external, personal traits can also process emotions in different cognitions [38].

The C-A-C framework has been used as a basic theory in numerous past studies across different contexts. Dai et al. [39] developed the applicability of the C-A-C framework to the behavior of social media users from a cognitive-affective-conative perspective; Hsiao [40] used the C-A-C framework to investigate online content sharing behavior, describing Internet users’ perception of the internet, their emotions about the internet, and the intentions of these users when using the technology (i.e., online content sharing).

**Figure 1:**
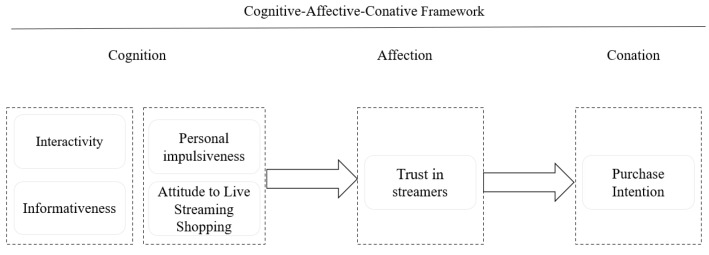
Conceptual framework.

The present study applies a cognitive-affective-conative framework to explain the relationship between interactivity, product information, personal impulse, attitude towards livestreaming shopping, trust in the streamer, and purchase intention in the context of livestreaming commerce (see Figure 1).

Consumer behavior typically consists of cognitive, affective, and conative aspects [41], although there are many studies that adopt a cognitive-affective-conative framework in the context of e-commerce [42,43,44]. Nonetheless, studies that account for consumers’ consumption behavior in livestreaming scenarios are rare. In the context of livestreaming shopping, cognitive factors are made up of consumers’ internal influences (personal impulses and attitudes towards livestreaming shopping) and external influences (interactivity and product information). Based on the fact that trust is related to affective factors, trust in the streamer is used as an effective factor. Conative factors consist of purchase intention. Figure 1 presents the following framework.

## 3. Hypotheses Development

### 3.1. The Effect of Streamer Trust on Purchase Intention

Trust in a streamer refers to the belief that the streamer can provide quality service and will not exploit and harm the customer [21,45]; it is formed through the emotional bond between the audience and the streamer [46]. As the leader of the livestreaming room, once the audience believes that the streamer is an expert in certain fields or that the streamer can meet their needs, the audience will be willing to continue watching the livestream and will seek help from the streamer [30]. During the livestream, audiences can send a bullet screen to ask questions or express their thoughts, and this continuous interaction allows them to develop a greater sense of trust in the streamer [47].

Streamer trust has a positive effect on transactions. Trust can help consumers reduce risk perception when dealing with streamers, encourage them to interact with streamers, and can directly affect their willingness to buy [20,48]. Therefore, the following hypothesis is proposed:

**H1.**  *Trust in streamers is positively associated with consumers’ purchase intentions.*

### 3.2. The Effect of Interactivity on Streamer Trust

Interactivity is seen as a key element of online communication and is a distinctive feature of livestreaming [22,49,50]. Compared with the traditional e-commerce shopping model, live streaming shopping is far more interactive [51], as audiences can directly ask questions to the streamer through the bullet screen, and the streamer can then provide rapid feedback to meet the audience’s needs [52].

Timely interactions also contribute to a high level of trust in the streamer among audiences [21]. Streamers’ timely answers to audience questions improved the customer service experience [30] and shortened the psychological distance between both parties [53]. Timely interactions also allow audiences to understand the product better and generate trust in the streamer [54]. Therefore, we infer that interactivity can increase trust in the streamer.

**H2.**  *Interactivity is positively associated with streamer trust.*

### 3.3. The Effect of Product Information on Streamer Trust

Livestreaming commerce provides consumers with comprehensive product information. Product information, such as access to details about a product’s performance and function, is the core element that drives purchasing behavior [27]. In a livestream, streamers can instantly understand the consumer’s demand for the product and adjust the presentation method according to the real-time feedback of the audience. Instead of finding the information they need from a large number of words and images on the website, consumers can directly get an accurate response from the streamer.

In traditional e-commerce, consumers are invisible to sellers [55] and can only view product information through text, pictures, and videos uploaded by merchants, which makes communication and the establishment of a trust relationship [56]. In livestreaming shopping, products are displayed through livestream, and audiences can better understand the products and streamers through visual communication. This effectively shortens the distance between the audience, the streamer, and the product [57]; as a result, the audience’s trust in the streamer also increases [30]. Therefore, we infer that the provision of product information can increase trust in the streamer.

**H3.**  *Product information is positively associated with streamer trust.*

### 3.4. The Effect of Personal Impulses on Steamer Trust

A trait is a temporally stable, cross-situational individual difference. One characteristic of an individual is a series of influences on responses associated with that trait, which determines their cognitive, affective, and conative styles [58]. Personal impulse as a personal trait entails the likelihood that consumers experience spontaneous impulses to buy and, thereby, act on these impulsive feelings with little attention to the consequences [59]. In previous studies, researchers who studied the moderating effects of individual impulses on cognitive states (e.g., website quality and information quality) and affective states (e.g., number of “likes”) provided some insights, and this study focused on the relationship between personal impulses and streamer trust. The interaction between the environment and individual impulses affects consumer behavior [26]. This is particularly the case when consumers purchase goods via livestreaming. Personal impulses increase the trust of the streamer and encourage them to participate in online transactions, thus forming a connection that directly affects purchase intention. Therefore, we make the following hypothesis:

**H4.**  *Personal impulse has a positive effect on streamer trust.*

### 3.5. The Effect of Attitudes towards Livestreaming Shopping on Streamer Trust

Attitude towards livestreaming shopping refers to people’s favorable impression of livestreaming shopping without considering the benefits or value of the experience. Chen and Lin [60] find that entertainment has a greater impact on attitude. That is, as the interaction between audiences and streamers increases, so too does the audience’s positive attitude towards livestreaming, and audiences will, thereby, develop trust in the streamer [61,62]. Therefore, we make the following hypothesis:

**H5.**  *The attitude towards Livestreaming shopping is positively associated with streamer trust.*

### 3.6. Moderated Effect of Livestreaming Value

“Livestreaming value” is an overall assessment of the subjective and objective factors that constitute a livestreaming shopping experience [63]. Given that livestreaming is not yet widespread or commonly used, consumers who participate in livestreaming may perceive it as an innovative approach [64]; this innovative quality represents the value of livestreaming. Livestreaming can provide practical value (authenticity, responsiveness, and visualization) [21], although, from the perspective of the responsiveness provided by the livestreaming, the audience in the livestreaming can enhance the trust of the streamer in the interaction with the streamer. However, the value of livestreaming may make the audience calmer, thus weakening the impact of interactivity on the streamer’s trust. The perceived authenticity and visualization of livestreaming enable audiences to better understand product information, which enhances the impact of product information on the trust of the streamer.

Considering personal impulse as a trait of consumers, some studies have found [65] that the impulse shopping tendency varies from consumer to consumer; consumers in a livestream may increase their trust in the streamer, which affects their purchase intention. However, under the influence of the value of the livestream, the role of personal impulse on streamer trust may be weakened.

Streamers provide product information, actively answer audiences’ questions, and gain their trust during livestreams [19,66], thereby altering the perception and attitude of the audience [1]. This change in attitude also affects trust in streamers, and we speculate that the value of livestreaming will strengthen the role of attitude towards livestreaming shopping on streamer trust. Therefore, the following hypotheses are proposed:

**H6.**  *The livestreaming value will weaken the effect of interactivity on streamer trust.*

**H7.**  *The livestreaming value will increase the effect of product information on streamer trust.*

**H8.**  *The livestreaming value will weaken the effect of personal impulse on streamer trust.*

**H9.**  *The livestreaming value will increase the effect of attitude towards livestreaming shopping on streamer trust.*

**Figure 2:**
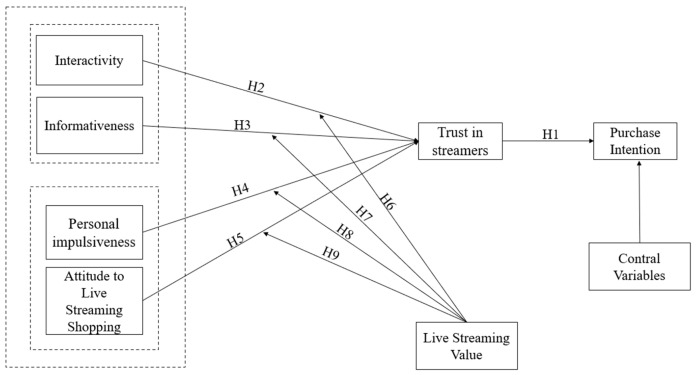
Research model.

Based on these hypotheses, the theoretical model of this study is shown in Figure 2. We also control for the potential impact of consumer gender, how often they watch the livestream, and the time spent livestreaming shopping.

## 4. Methods

To validate the conceptual model constructed based on theoretical understandings, we conducted a questionnaire survey to collect empirical data. The measurement, data collection process, and final sample analysis details are discussed next.

### 4.1. Measurement Items

Seven variables, namely interactivity (INT), information (INF), purchase intention (PIN), attitude towards livestreaming shopping (ALSC), livestreaming value (LSV), trust in streamers (TS), and personal impulse (PI), are included in this study. The control variables include demographic information, such as gender, how often individuals watch livestreams, and the time spent on livestreaming shopping. Since the purpose of this study is to explore the factors that influence audiences’ willingness to purchase during livestreaming, we modified and adjusted the questions to fit the scenario of the livestreaming. We used a seven-point Likert scale, ranging from “1 = strongly disagree” to “7 = strongly agreed”. All questions were designed in line with the extant literature. The measurement items and related sources are shown in Table 2.

### 4.2. Samples and Procedures

This study investigated 385 college students of different majors and grades from universities located in Hangzhou, China. We invited a professional streamer to demonstrate the product and the interaction between the streamer and audiences through Ding Talk, all of which was conducted in real time in the live room. In order to improve the authenticity of the questionnaire answers, we used a paper questionnaire. According to the setting of the reverse question, 24 invalid questionnaires were excluded, for a total of 361 valid questionnaires. The sample structure is shown in Table 3. The sample consisted of more women (64.9%) than men (35.1%), with more than half of respondents (57.6%) rarely or occasionally watching livestreams, and more than half (51%) used livestreaming to shop for less than a month.

### 4.3. Data Analysis and Results

The data was analyzed using SPSS 26.0 (IBM: Armonk, NY, USA) which was responsible for descriptive data analysis and co-method bias testing; we adopted the partial least squares (PLS) method and used SmartPLS 4 (SmartPLS: Oststeinbek, Germany) to evaluate the measurement model and structural model. The partial least squares equation modeling is suitable not only for working with atypical distribution data but also for samples that are smaller than 500 [69]. Based on a two-step data analysis process, we first conducted the assessment of the measurement model with the evaluation of reliability, convergent validity, and discriminant validity, and then we tested the structural model to evaluate the hypotheses.

### 4.4. Measurement Model

We evaluated the reliability of the constructs with Cronbach’s alpha (α) and composite reliability (CR). As shown in Table 4, Cronbach’s alpha (α) for all concepts ranged from 0.716 to 0.947, and the composite reliabilities were all above 0.7, which means a favorable reliability [70,71]. In addition, we evaluated convergent validity using the factor loadings and the average variance extraction (AVE). As shown in Table 4 and Table 5, all factor loadings were more than 0.6, and all AVEs were above 0.5, indicating satisfactory convergence validity [70].

According to the Nunnally and Bernstein [71] recommendation, we contrasted the square roots of the AVEs with construct correlations. The correlation coefficient matrix in Table 6 shows that the square root of AVE for each dimension is greater than the correlation coefficient between dimensions. Therefore, each dimension of this study has sufficient discriminatory properties with other dimensions, indicating good discriminant validity.

### 4.5. Structural Model and Hypotheses Testing

**Figure 3:**
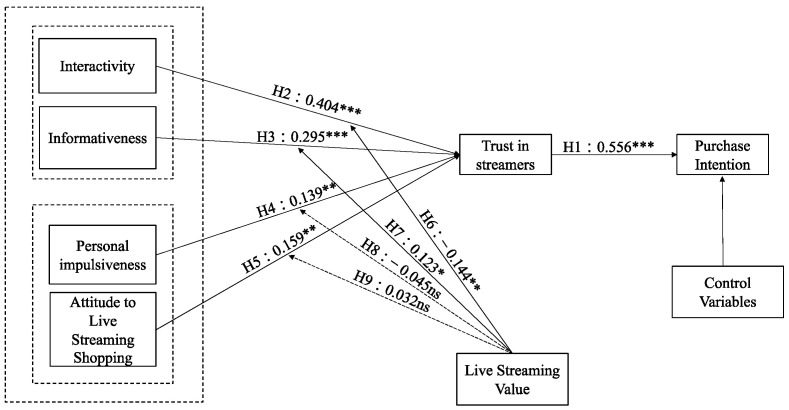
Hypothesis test results. * *p* < 0.05, ** *p* < 0.01, *** *p* < 0.001, ns: not significant at the 5% significance level.

In order to obtain stable and reliable results, we used the bootstrapping algorithm of SmartPLS 3.2.9 (SmartPLS: Oststeinbek, Germany) to run 5000 times to study the path coefficient and significance of each hypothesis. The hypothesis test results are shown in Table 7 and Figure 3. Firstly, regarding cognition and affection, interactivity, information, personal impulse, and attitude towards livestreaming shopping, each had a positive effect on streamer trust (β = 0.404, *p* < 0.001; β = 0.295, *p* < 0.001; β = 0.139, *p* < 0.01; β = 0.159, *p* < 0.01), indicating support for H2, H3, H4, and H5. Secondly, regarding the relationship between affection and emotion, streamer trust was shown to have a positive effect on purchase intention (β = 0.556, *p* < 0.001), supporting H1. Finally, regarding the moderating effect of livestreaming value, this study uses a four-stage PLS method to test the interaction effect of streamer trust (i.e., interactivity × streamer trust, information × streamer trust, personal impulse × streamer trust and attitude to livestreaming shopping × streamer trust), the results show that livestreaming value can significantly weaken the relationship between interactivity and streamer trust (β = −0.144, *p* < 0.01), and that livestreaming value can significantly enhance the relationship between information and streamer trust (β = 0.123, *p* < 0.01). However, it does not significantly weaken the relationship between personal impulse and streamer trust (β = −0.045, *p* > 0.05), and it does not significantly enhance the relationship between attitude towards livestreaming shopping and streamer trust (β = 0.032, *p* > 0.05), which means that H6 and H7 are supported, and H8 and H9 are not supported for hypothesis test results.

## 5. Discussion

As an important part of e-commerce, livestreaming has created huge benefits for livestreaming platforms, brands, streamers, and audiences. However, the streamer’s trust will reduce the audience’s willingness to buy products or generate positive word of mouth. This will harm the interests of all parties and hinder the sustainable development of livestreaming commerce [72]. Therefore, it is of great significance to study how consumers increase their willingness to buy under the influence of streamer trust. This study adopts the C-A-C framework to explore the form of consumer-streamer to study consumers’ purchase intention in livestreaming. Through a questionnaire survey of 385 respondents under the simulation of the livestreaming scene of a professional streamer, we found that consumers’ external influence and their own internal influence can affect the audience’s emotional trust in the streamer differently, which will encourage or prevent the audience from rewarding her/him by generating positive public praise. 

The empirical results verify most of our hypotheses and demonstrate the applicability of the C-A-C model in livestreaming commerce research. Firstly, the results show that streamer trust has a positive effect on purchase intention, and enhancing streamer trust is also an important and effective measure to trigger purchase intention, a finding that is consistent with past research [20,30,73]. Thus, audiences in livestreaming rooms need to have a considerable degree of trust in the streamer before making the decision to purchase a product. As external influences on cognitive factors, interactivity, and product information have a positive impact on streamer trust. This means that positive interactions with streamers can increase audience trust. If audiences trust streamers, then they, by extension, trust that the streamers have the ability to introduce and evaluate products reliably, accurately reflecting the consumer’s preferences and needs [20]. In the livestreaming process, information is the core element that drives the audience’s product purchases. The audience seeks to meet their need for information via livestreaming, but the uncertainty of product information often leads to a complex online shopping process. The reduction of information asymmetry between audiences and products reduces the likelihood that audiences will suffer financial losses due to perceived quality differences [27]. More specific product information can increase audiences’ trust in streamers. From the perspective of internal influences on cognitive factors, personal impulses and attitudes toward livestreaming shopping also have a positive impact on streamer trust. The external and internal influences that constitute cognitive factors are also involved in a process in which the audience in the livestreaming room receives and processes information. In doing so, they build trust with the streamer, which is an effective factor, and finally arrive at a willingness to buy, which is an audience behavior that stems from cognition and affection.

Although most of the hypotheses proposed in this paper have been supported, several have not been supported. The results show that the value of livestreaming can significantly weaken the relationship between interactivity and streamer trust, and it can also significantly enhance the relationship between information and streamer trust. However, it does not significantly weaken the relationship between personal impulse and streamer trust, although this may be because the participants in this study are relatively less impulsive. We also found that livestreaming value does not significantly enhance the relationship between the attitude towards livestreaming shopping and streamer trust, which indicates that livestreaming value may not affect the impact of attitude towards livestreaming shopping on streamer trust.

## 6. Implications

### 6.1. Theoretical Contributions

Our research provides several important theoretical contributions. First, our research contributes to the literature on livestreaming commerce by developing a theoretical model that reveals how livestreaming commerce affects consumers’ purchase intentions from the perspectives of interactivity, product information, personal impulse, attitudes towards livestreaming shopping, livestreaming value, and streamer trust. The previous literature has also explored many reasons why consumers buy, including external influences [1,27,29], internal influences [25,26,27], and streamer trust [21,30]. Prior works, however, did not combine external and internal influences with streamer trust. This study, therefore, fills this gap within the context of livestreaming commerce.

Furthermore, the C-A-C framework was developed to understand the causal relationship between individual cognition, affection, and conation [41]. Although there are studies that adopt the cognitive-affective-conative framework in the context of e-commerce [42,43,44], there is currently no precedent for applying the C-A-C framework to livestreaming commerce scenarios. Thus, this research expands the application of the C-A-C framework and applies it to a new, important phenomenon. As expected, the cognition of audiences in the livestream room (i.e., internal and external influences) showed a significant impact on an individual’s affection (i.e., trust in the host), thus influencing their conation (i.e., purchase intentions). Given this connection, we believe that the C-A-C framework is a highly beneficial model for understanding the impact of individual cognitive factors on their affective, conative movements in many cases. Therefore, the C-A-C framework should be used and tested in other environments in the future.

The results and theoretical application of this research promote the theoretical development of the field of livestreaming commerce and lay the foundation for future research to enhance our understanding of consumer behavior in livestreaming shopping.

### 6.2. Practical Significance

The study also provides some actionable guidelines for practitioners in livestreaming commerce. Firstly, as an opinion leader and source of information, streamers should pay attention to building trust with their audiences during the livestream process, as streamer trust is positively associated with purchase intention. Streamers should also interact more with their audience in the livestream rooms, which affects their trust relationship with viewers. Moreover, the streamer should respond to the audience’s questions in a timely fashion, show the audience goods in a thorough way and provide specific product information. This can effectively reduce audiences’ doubts about the product and increase their trust in the streamer. At the same time, managers should systematically train streamers to improve their skills in terms of showcasing goods in a livestream and enhancing their interactions with the audience. Trust in the streamer will directly transfer to the product. It is, therefore, recommended that managers pay attention to the streamers with a good reputation and a high level of trust with their audience, as these individuals can foster a positive shopping experience and attract more consumers.

Moreover, for consumers, there is a noteworthy emotion (i.e., personal impulse) when purchasing goods in a livestream room. Our research shows that personal impulse can have a positive effect on streamer trust, which may lead to consumer purchasing behavior. The ability of consumers to control personal impulses is an effective way to avoid impulse purchases that can lead to wasted money. At the same time, consumers’ attitudes towards livestreaming shopping also are positively associated with the streamer’s trust, and managers should properly guide the streamer in terms of how to interact correctly with their audience. Doing so will help improve the audience’s positive attitude toward the livestreaming and increase their trust in the streamer.

Finally, we found that while the value of livestreaming can significantly enhance the relationship between information and streamer trust, it can also significantly weaken the relationship between interactivity and streamer trust. Thus, the streamer should still focus on the product information and should display the item in a way that helps the audience experience it as they would in person. 

## 7. Conclusions

This study shows that there is a dynamic mechanism between consumers and streamers. The purchase behavior of consumers in the livestream room is the process from cognition to affection and then to conation. It is the behavior of consumers in the livestream room based on cognition and affection. Our research shows that streamer trust provides an opportunity for the generation of purchase intention, which is consistent with Zhao’s research conclusion [74]. However, consumers’ cognitive factors are also the most important. On the basis of the C-A-C framework, cognitive factors directly affect affectional factors, and consumers’ external and internal influences should also be paid attention to. 

However, the current research has some limitations. Firstly, our research subjects are college students whose ages are concentrated in the 18–25 years old range. Future research will increase the age diversity among subjects. Furthermore, our research background is limited to livestreaming commerce; future research should adjust and modify the existing research model and apply it to other scenarios. Second, our sample is derived from Chinese livestreaming commerce participants, which may limit the findings. Because China’s livestreaming commerce is more developed than other countries, most of the relevant research in this field is also focused on the Chinese context.

Nevertheless, future studies could consider livestreaming users in other countries to verify whether the current research model has good external validity. The important role of digital technology in purchase intention should also be valued.

## Figures and Tables

**Table 1:** Literature review and summary related to livestreaming commerce.

Literature	Internal Impact	External Influence	Attitude	Trust	Theory	Context
Zhang et al. [25]	√	√	×	×	Affective-cognitive framework	Livestreaming
Wu et al. [26]	√	√	×	×	Competitive arousal model	Online shopping
Chen et al. [27]	√	√	×	×	Dual-process theory	Livestreaming
Wongkitrungrueng et al. [28]	×	√	×	×	Sales orientation and online relationship marketing	Livestreaming
Kang et al. [29]	×	√	×	×	S-O-R theory	Livestreaming
Wongkitrungrueng and Assarut [21]	×	√	×	√		Social commerce
Lu et al. [31]	×	√	×	√	Social presence theory	Social commerce
Lu and Chen [20]	×	√	×	√	Signaling theory	Livestreaming
Park and Lin [32]	×	√	√	√	Match-up hypothesis	Livestreaming
Zhang et al. [30]	√	√	×	√	Socio-technical system theory	Social commerce
This Study	√	√	√	√	C-A-C framework	Livestreaming

**Table 2:** Measurement items for the constructs.

Constructs	Items	Sources
Interactivity	1. The streamers actively responded to viewers’ questions.	Ma et al. [67]
	2. The streamers answered viewers’ questions and requests in time.	
	3. The streamers provided relevant information in response to viewers’ inquiries.	
Informativeness	1. The livestreaming process supplies relevant information on products.	Adapted from Firat [68]
	2. The livestreaming process provides timely information on products.	
	3. The livestreaming tells people about products when they need the information.	
Personal Impulsiveness	1. I often buy things without thinking.	Wu et al. [26]
	2. “I see it, I buy it” describes me.	
	3. “Just do it” describes the way I buy things.	
Attitude to Livestreaming Shopping	1. I think watching a livestream is a good idea.	Chen and Lin [60]
	2. My attitude toward watching livestreams is positive.	
	3. I like watching livestreams.	
Livestreaming Value	1. Livestreaming is valuable.	Adapted from Firat [68]
	2. Livestreaming is useful.	
	3. Livestreaming is important.	
Trust in Streamers	1. I believe in the information that the streamer provides through livestreaming shopping.	Zhang et al. [30]
	2. I can trust the streamer on livestreaming shopping.	
	3. I believe that the streamer on livestreaming shopping is trustworthy.	
Purchase Intention	1. I am very likely to buy the products from livestreaming shopping.	Lu and Chen [20]
	2. I would consider buying the products from livestreaming shopping in the future.	
	3. I intend to buy the products from livestreaming shopping.	

**Table 3:** Sample statistical structure.

Measure	Items	Frequency	Percent
Gender	Female	127	35.1%
	Male	234	64.9%
Frequency	Rarely	208	57.6%
	Sometimes	104	28.8%
	Usually	49	13.6%
Time of using live shopping	Less than 1 month	184	51%
	1–12 months	80	22.1%
	1–2 years	66	18.3%
	More than 2 years	31	8.6%

**Table 4:** Results of reliability and validity analysis.

Constructs & Item		Cronbach’s α	CR	AVE
Interactivity (INT)	INT1	0.947	0.966	0.904
	INT2			
	INT3			
Informativeness (INF)	INF1	0.851	0.910	0.771
	INF2			
	INF3			
Purchase Intention (PIN)	PIN1	0.882	0.927	0.809
	PIN2			
	PIN3			
	PIN4			
Attitude to Livestreaming Shopping (ALSC)	ALSC1	0.821	0.888	0.725
	ALSC2			
	ALSC3			
Livestreaming Value (LSV)	LSV1	0.716	0.840	0.637
	LSV2			
	LSV3			
Trust in Streamers (TS)	TS1	0.881	0.926	0.807
	TS2			
	TS3			
Personal Impulsiveness (PI)	PI1	0.759	0.860	0.672
	PI2			
	PI3			

**Table 5:** Validity of questions.

Question	Factor						
	1	2	3	4	5	6	7
INF1	0.214	0.192	**0.768**	−0.084	0.263	0.052	0.169
INF2	0.228	0.247	**0.753**	−0.086	0.151	−0.038	0.173
INF3	0.161	0.270	**0.724**	0.012	0.380	0.009	0.160
PI1	0.049	0.044	−0.164	−0.085	0.206	**0.828**	0.055
PI2	0.045	0.095	0.032	0.140	−0.147	**0.800**	−0.062
PI4	0.053	0.056	0.130	0.145	−0.034	**0.809**	0.039
ALSC1	−0.027	0.007	−0.051	**0.863**	−0.073	0.076	0.206
ALSC2	0.070	0.014	0.006	**0.861**	−0.007	−0.050	0.144
ALSC3	0.101	0.106	−0.088	**0.785**	−0.048	0.210	0.063
TS1	0.190	**0.827**	0.245	0.073	0.120	0.114	0.088
TS2	0.239	**0.799**	0.184	0.039	0.245	0.052	0.091
TS3	0.211	**0.766**	0.225	0.070	0.238	0.111	0.249
LSV1	0.077	0.061	0.163	0.097	0.400	0.033	**0.694**
LSV2	0.104	0.191	0.087	0.150	−0.017	−0.062	**0.813**
LSV3	0.120	0.080	0.164	0.210	0.024	0.065	**0.716**
PIN1	**0.794**	0.137	0.246	0.077	0.200	0.041	0.161
PIN2	**0.794**	0.245	0.207	0.097	0.207	0.049	0.177
PIN3	**0.861**	0.226	0.109	0.038	0.184	0.103	0.021
INT1	0.349	0.282	0.334	−0.086	**0.737**	0.005	0.151
INT2	0.299	0.327	0.376	−0.087	**0.724**	−0.022	0.102
INT3	0.344	0.292	0.366	−0.111	**0.710**	−0.011	0.099

**Table 6:** Results of correlation analysis and discriminant validity tests.

	ALSC	INF	INT	LSV	PI	PIN	TS
ALSC	**0.851**	–	–	–	–	–	–
INF	−0.047	**0.878**	–	–	–	–	–
INT	−0.093	0.732	**0.951**	–	–	–	–
LSV	0.312	0.433	0.386	**0.798**	–	–	–
PI	0.196	0.050	0.047	0.067	**0.820**	–	–
PIN	0.158	0.536	0.631	0.361	0.157	**0.899**	–
TS	0.145	0.597	0.628	0.398	0.198	0.555	**0.898**

**Table 7:** Hypothesis test results.

Main Effects Results	Path Coefficients	Standard Deviation	*t* Value	*p* Value	Hypothesis
TS→PIN	0.556	0.040	14.050	0.000	H1 was supported
INT→TS	0.404	0.067	5.995	0.000	H2 was supported
INF→TS	0.259	0.077	3.815	0.000	H3 was supported
PI→TS	0.139	0.041	3.359	0.001	H4 was supported
ALSC→TS	0.159	0.051	3.105	0.002	H5 was supported

## Data Availability

The data presented in this study are available on request from the corresponding author.
